# Dynamic malaria hotspots in an open cohort in western Kenya

**DOI:** 10.1038/s41598-017-13801-6

**Published:** 2018-01-12

**Authors:** Alyssa Platt, Andrew A. Obala, Charlie MacIntyre, Barasa Otsyula, Wendy Prudhomme O’ Meara

**Affiliations:** 1 Duke Global Health Institute, Durham, North Carolina United States of America; 2 Department of Biostatistics and Bioinformatics, Duke University, Eldoret, North Carolina United States of America; 3 College of Health Sciences, Moi University, Eldoret, Kenya; 4 Campbell University School of Osteopathic Medicine, Buies Creek, North Carolina United States of America; 5 Department of Medicine, Duke University, Durham, North Carolina United States of America

## Abstract

Malaria hotspots, defined as areas where transmission intensity exceeds the average level, become more pronounced as transmission declines. Targeting hotspots may accelerate reductions in transmission and could be pivotal for malaria elimination. Determinants of hotspot location, particularly of their movement, are poorly understood. We used spatial statistical methods to identify foci of incidence of self-reported malaria in a large census population of 64,000 people, in 8,290 compounds over a 2.5-year study period. Regression models examine stability of hotspots and identify static and dynamic correlates with their location. Hotspot location changed over short time-periods, rarely recurring in the same area. Hotspots identified in spring versus fall season differed in their stability. Households located in a hotspot in the fall were more likely to be located in a hotspot the following fall (RR = 1.77, 95% CI: 1.66–1.89), but the opposite was true for compounds in spring hotspots (RR = 0.15, 95% CI: 0.08–0.28). Location within a hotspot was related to environmental and static household characteristics such as distance to roads or rivers. Human migration into a household was correlated with risk of hotspot membership, but the direction of the association differed based on the origin of the migration event.

## Introduction

A hotspot of malaria transmission is defined as ‘a geographical area within an endemic focus of malaria transmission where transmission intensity exceeds the average level’^1^. Hotspots have been identified at many spatial scales, from groups of households to multi-country regional zones, using many different indices of malaria burden. It has been noted that although hotspots occur across the spectrum of transmission intensity, such spatial heterogeneity is more pronounced in areas of lower transmission and uneven rainfall.

Interventions that are designed to leverage the spatial heterogeneity of transmission are at the forefront of the operations research agenda. Identifying hotspots could significantly improve targeting of control measures which could be more effective than spreading equivalent resources over larger areas or populations^2^. Furthermore, hotspots have been postulated to act as reservoirs of residual transmission in areas with otherwise effective control measures and may ‘fuel’ continual transmission within a larger area. If this is true, then interventions directed at hotspots could reduce transmission outside of the targeted area.

Targeting hotspots requires identifying, or ideally predicting, hotspot locations. Identifying hotspots has become an active area of investigation and several studies have shed light on the possible factors that contribute to foci of transmission, including environmental conditions^3–7^, behavioral factors^4,7,8^, and previous exposure and acquired immunity^1,9,10^. Understanding the correlates of hotspot location may help to predict hotspots.

The majority of hotspot studies have used a cross-sectional approach and analyzed data over a limited time period^7,11–13^. A handful of studies that investigated febrile hotspot stability over longer periods of time have reported conflicting results^5,9,10^. Hotspots identified in longitudinal cohort studies can be stable, usually related to fixed environmental risk factors, or may move from year to year and season to season^5,9,10,12,14–18^. Why they move and whether there is pattern to the movement is unknown. What is certain is that predicting future hotspots is not as straightforward as identifying current hotspots.

Using data from a large, population based cohort in western Kenya, we first determine hotspot location, then quantify the spatial and temporal stability of malaria hotspots and identify both static and dynamic correlates of hotspot location.

## Methods

### Study area

The Webuye Health and Demographic Surveillance System (WHDSS) was established in 2007 in the Webuye Division of Bungoma East District, western Kenya as an activity of the Moi University and Vlaamse Interuniversitair Raad – University Development Cooperation (Belgium) (VLIR-UOS) Health Sciences Project as a natural progression of Moi University’s Community Based Education and Service (COBES) program. The WHDSS encompasses an area of 115 km^2^. Approximately 52% of the population lives below the poverty line, with limited access to piped water and electricity. The main economic activities include sugarcane farming as a cash crop, while maize, sorghum and millet are cultivated as subsistence crops. Dairy and poultry farming are also widely practiced. The WHDSS is described in more detail elsewhere^19^.

The area has a tropical climate and lies at an elevation of 1523 m above sea level. It experiences an average annual temperature of 24 °C, and receives high rainfall ranging from 1200 to 1800 mm annually. Malaria burden in Bungoma East and the WHDSS is high, due to the suitable climate^20^. Malaria is perennial with peaks following the rains. Typically, transmission peaks in April-June following the long rains with a smaller peak in October following the short rains.

### Data collection

All households in the WHDSS were mapped and enrolled at baseline. Baseline data collection occurred in November 2008 and continued for 4 months. Data collection was repeated biannually in the fall from September 1 to December 31 and in the spring from March 1 – June 30th. Each data collection period is called a ‘Round’. GPS coordinates were recorded for each compound, consisting of between 1–16 households. A household is defined as a group of people who regularly “eat from the same pot” (in other words, share cooking arrangements). A “resident” of a household is defined as an individual who has lived in that household continuously for a period of at least four calendar months prior to the interview date^21^. Households within a compound usually consist of extended family in separate but closely grouped dwellings. Twice per year, enumerators visited every household to record all births, deaths, illnesses and migrations. Data were either collected on paper forms and entered into a database or collected on personal data assistants using Pendragon forms.

The WHDSS has 6 sublocations (Median area = 20.0 sq km) and within each sublocation are approximately 140 villages. Due to inconsistencies in data collection in one survey round, one sublocation in the southernmost part of the study area was removed from the analysis. Although teams moved from village to village within a sublocation, several teams of data collectors worked simultaneously in all parts of the WHDSS area at once during each round of data collection.

### Data processing

#### Malaria Morbidity

Information about all illnesses experienced by household members since the last study visit were self-reported (in the case of minor children, reported by a guardian). Recall windows are therefore the amount of time elapsed since the previous study visit (estimated median recall window is 182 days). Dates of illness and treatment were recorded. All illness episodes that were self-reported as ‘malaria’ (ICD-10-CM codes B54, “Unspecified Malaria”) were included in the analysis. To avoid double-counting the same illness, any malaria episode that occurred within 3 weeks of an initial malaria illness was censored. To capture compound-level malaria incidence by season, malaria episodes were aggregated to the compound level and further aggregated by 6-month periods (Spring: January-June; Fall: July-December, each interval centered on a ‘Round’ in April and September)(Table 1). We required that an individual be present within a compound for the entirety of the 6-month interval for their reported malaria episode to be counted for that compound. The recall period of morbidity data in the baseline round (Round 1), were not consistent with subsequent rounds, therefore, our analysis period begins with Round 2.

**Table 1:** Descriptive statistics of the study population (n (%) unless otherwise noted).

Observations	8300
Number (%) with data by rounds (Total Population)	
All rounds	7409 (89.4%)
Missed 1	653 (7.9%)
Missed 2 or more	238 (2.9%)
Number (%) with data by rounds (Children 0–5 years)	
Households with child under 5 in any round	6606 (79.6%)
Households with child under 5 in all rounds	4591 (55.3%)
Altitude (meters), median (IQR)	1505 (1475, 1559)
Compound population (Mean), median (IQR)	7 (5, 9.6)
Household head or spouse in compound farming^1^	6212 (80.4%)
Number of cattle owned^1^, median (IQR)	1 (0, 3)
Number of sheep/goats owned^1^, median (IQR)	0 (0, 1)
Number of poultry owned^1^, median (IQR)	4 (1, 8)
Land ownership (acres)^1^, median (IQR)	1.25 (0.64, 2.75)
Water source^1,2^	
Piped	2889 (37.3%)
Spring (protected or unprotected)	2841 (36.7%)
Well	1141 (13.7%)
Other	1113 (14.4%)
Distance to river (meters), median (IQR)	2807 (1411, 3921)
Distance to road (meters), median (IQR)	267 (117, 485)
Distance to health facility (meters), median (IQR)	2771 (1859, 3748)

^1^Household characteristics (N = 7713). ^2^Multiple water sources per compound are possible.

Representing 20% of the total population in the WHDSS, children age 0 to 5 account for 40% of the malaria cases reported. Globally, children age 0 to 5 are a high risk group for infection and morbidity due to malaria illness^22^, therefore, we performed all analyses on this subgroup in addition to the full census population.

#### Definition of Hotspots

Hotspots were defined spatially using the Kuldorff spatial-only scan statistic^23^. This approach to identifying hotspots has been used in previous research^1,4,6,7,9,11,12^. The scan statistic is calculated using a discrete Poisson model with an outcome of the count of malaria episodes per compound, per 6-month interval. Spatial scan test statistics are calculated by moving a circular window systematically through the study space of a geographic area and varying the radius of the window. For each location and radius, observed cases are compared to expected cases under the null hypothesis that the pattern of locations of malaria cases is proportional to the population at risk, against an alternative that, in some locations, malaria cases will exceed the number that would be expected under the null model. That is, if the rate of malaria cases throughout the study area is λ (where $\lambda =\tfrac{Total\,malaria\,cases}{Total\,population\,at\,risk}$), then we would expect the rate to be λ both inside and outside of any particular window within the study area. A maximum likelihood ratio test evaluates whether the null or alternative model better fits the data at each location. The scan statistic is the maximum observed likelihood ratio statistic over all possible window sizes with a p-value obtained through Monte Carlo simulation based on discrete Poisson randomization. P-values are adjusted to account for multiple testing across all potential clusters. In our analysis, we considered a p-value < 0.05 to indicate the presence of a malaria hotspot. Because foci of malaria fever incidence are generally 1–2 km in size^1^, we limited the size of our window to a 2 kilometer radius and limited the percentage of population-at-risk that could fall into a single hotspot as 30%^9^. The population at risk included all individuals present in a compound. For analyses of the subpopulation of children age 0 to 5, we limited the population at risk to individuals in this age group. By extension, only compounds that had at least one child in this age group during a particular round could be included in calculation of the scan statistic.

Malaria reporting rates can vary over time due to the seasonality of transmission. Data collection rounds lasted for several months, therefore the recall window for a household may fall within different parts of the malaria season from other households leading to differing recall lengths between households. To account for this, we performed covariate adjustment prior to computing spatial scan statistics by entering median village visit dates for a particular round into exponential models to compute predicted counts of malaria episodes at the compound level for each interval. Predicted values then replaced the population offsets to yield a visit date adjusted spatial scan statistic^24^.

Scan statistics were computed using spatial-only models and were repeated for every 6-month interval over the course of each of the five included study rounds. Relative risks of observed versus expected cases were computed for each identified cluster.

#### Static Correlates with Febrile Hotspot Location

We investigated compound socioeconomic characteristics plausibly correlated with the binary outcome of location within a hotspot. In the WHDSS data, household socioeconomic variables are only available at enrollment, however we expect such characteristics to change little over time. Compound socioeconomic characteristics used include: maximum compound population, whether the compound had household heads (or spouses) involved in farming as an income generating activity, number of cattle, number of sheep and goats, number of poultry, acres of land owned, use of piped water, use of water collected from a spring, use of well water, and use of other water sources.

Government health facilities in the WHDSS were mapped in 2007 and the list was updated in 2012 to include new facilities. Shapefiles containing locations of rivers were obtained from the International Livestock Research Group^25^. Euclidean distances to a river, a road, and the nearest health facility were calculated for each compound in WHDSS using ArcGIS 10.0 software.

#### Confounding with environmental conditions

Because we wished to isolate the effects of static household characteristics, transportation and health access, we controlled for confounding of vector habitat using remote sensing data. This included calculation and inclusion of a Topographic Wetness Index (TWI) (details in Supplemental File 1)^26^ and elevation (collected with GPS coordinate data). In addition, time series of data from the MODerate-resolution Imaging Spectro-radiometer (MODIS) sensors on-board NASA’s Terra satellite were extracted for each compound for the full calendar years during the study period from January 2009 to December 2011^27^. Temporal Fourier Analysis (TFA) was used to achieve temporal ordination of the variables as series of harmonics of different seasonal frequencies (detailed methods in Supplemental File 1)^28,29^. Mean, minimum, maximum, amplitude, phase, and sum of squared residuals were extracted for annul, biannual, and triannual cycles for four channels of daytime land surface temperature, night time land surface temperature, Middle Infrared Reflectance (MIR), and Enhanced Vegetation Index (EVI). A rigorous variable selection process (Supplemental File 1) was applied to reduce the resulting 72 parameters to 21 selected variables (Supplemental Fig. 1).

#### Dynamic human population and migration

Compound population was computed for each 6-month interval using dynamic migration, birth, and death datasets to adjust original household counts (Table 1). Additional dynamic variables of interest included number of births, number of migrations into a compound from an urban area, number of migrations into a compound from a rural area, number of migrations from a WHDSS household that was in a hotspot in a previous interval, and number of migrations from a WHDSS household that was not in a hotspot in the previous interval.

### Statistical analysis

The binary outcome of location within a hotspot at any point in time was analyzed using modified Poisson regressions by specifying generalized linear models with Poisson distributions, log link functions, and robust standard errors^30^. Modified Poisson regressions offer stable estimation of binary outcomes with an advantage of interpretation of exponentiated regression parameters as risk ratios. Covariates of interest were static demographic, geographic and socioeconomic characteristics of the compounds. All models were adjusted with the reduced set of Fourier analyzed remotely-sensed environmental variables. Parameter estimates are presented as relative risks (RR).

In order to explore stability of hotspots in time, we specified modified Poisson regressions for outcomes of current hotspot membership as well as lagged outcome variables for previous membership in a hotspot. Regressions were specified using generalized estimating equations (GEE) with exchangeable working correlation matrices, to adjusted for repeated measurements within a compound, and robust standard errors^31^. In the children age 0 to 5 sample, we allowed previous interval and 2 interval lagged hotspot membership to include both age 0 to 5 hotspots as well as total population hotspots. As with calculation of the scan statistic, only compounds with at least one child age 0 to 5 in a particular round could be included in the regression analysis of hotspot membership for that round. Separate models were specified for each sample by length of lag to reduce correlation between explanatory variables. Since data were collected biannually, we controlled for possible modification of the lagged effect by current season using a season by lag interaction term. Because a lagged covariate is used, the outcome variable in dynamic regressions applies specifically to four 6 month intervals over two calendar years from spring 2010 to fall 2011.

A second set of modified Poisson GEE regressions were specified to explore the effects of population dynamics on hotspot location. Dynamic covariates such as compound population at each interval, migration, and new births were included as covariates in these models. The outcome was location within a hotspot during a specific 6-month interval.

Because the goal of this study was exploratory and hypothesis-generating, we did not make adjustments for multiple comparisons. Alphas for all statistical tests were 0.05.

### Missing data

Missing data in the HDSS occurred through non-response, lack of geographic coordinates, and loss of information due to varying lengths of recall periods for illness. Non-response in the WHDSS was minimal (<5%) and therefore not expected to bias the data. Those who were missing compound coordinates were less likely to report malaria illness, however, we expect this type of missing data to be spatially homogenous and thus have little impact on our analysis of malaria hotspots. Finally, household/compound characteristics were missing for 7% of the compounds, which will not affect hotspot identification or correlation with dynamic variables but could potentially bias effect estimates in our static analysis. To evaluate this, we performed chi square tests on the age 0 to 5 and total population sample to evaluate potential dependence between the outcome (hotspot membership) and probability of missing data in compound characteristics and quantified the effect by calculating inverse probability weights using available spatially derived variables such as the TFA analytic variables, TWI, and altitude.

### Ethical Approval

The WHDSS received study approval from the joint Institutional Review and Ethical Committee (IREC) of Moi University and Moi Teaching and Referral Hospital (MTRH) and all methods were performed in accordance with the relevant guidelines and regulations established by IREC and MTRH. Verbal informed consent was obtained from the head of household prior to enrolment into the surveillance site. The site operated under direction of the Scientific Committee of Moi University-VLIR-OUS Health Sciences Project University Management through the Moi University VLIR UOS Steering Committee and worked closely with a Community Advisory Board.

## Results

### Demographics and location

A total of 8300 compounds comprising 12,602 households were included in the analysis. Only 10.7% of compounds missed a survey round, the majority only missing a single round (Table 1). Eighty percent of compounds had a child below five years of age in at least one 6-month interval. A median household had 7 individuals and more than 80% of compounds were involved in farming as an income generating activity. The average compound was greater than 2.5 km from the nearest river or health facility, but about a quarter kilometer from the nearest local road.

### Malaria in the study population

Incidence of malaria was highest in children age 0 to 5, ranging between 4.5 episodes per 100 in a six-month period (fall 2011) and a high of 7.1 (fall 2009; Table 2). Over this same period, malaria incidence in the entire population ranged from 1.8 to 3.6 episodes per 100 people.

**Table 2:** Counts and descriptive statistics for dynamic covariates.

**Variable**	**Fall 2009**	**Spring 2010**	**Fall 2010**	**Spring 2011**	**Fall 2011**	**All**
**All compounds**	8218	7812	8013	7931	8049	40,023
New births	915	855	860	859	790	4279
In-migrations from hotspot	—	3	2	16	21	42
In-migrations from non-hotspot	—	23	81	68	93	265
In-migrations from rural area^1^	212	135	335	293	405	1380
In-migrations from urban area^1^	98	71	125	111	167	572
Compound population, median (IQR)	7 (5,10)	7 (5,10)	7 (5,10)	7 (5,10)	7 (5,10)	
Children under 5 in compound, median (IQR)	1 (0,2)	1 (0,2)	1 (0,2)	1 (0,2)	1 (0,2)	
Total malaria cases	2292	1090	2065	1671	1261	8379
Total population	64324	61688	63370	62874	63858	316114
**Compounds with children <5yrs**	6032	5688	5721	5611	5588	28640
In-migrations from hotspot	—	3	2	14	16	35
In-migrations from non-hotspot	—	17	69	56	73	215
In-migrations from rural area^1^	168	102	273	226	286	1055
In-migrations from urban area^1^	79	55	96	95	135	460
Total malaria cases	875	449	810	683	501	3318
Total population	12385	11700	11636	11401	11196	58318

^1^outside of WHDSS.

**Figure 1:**
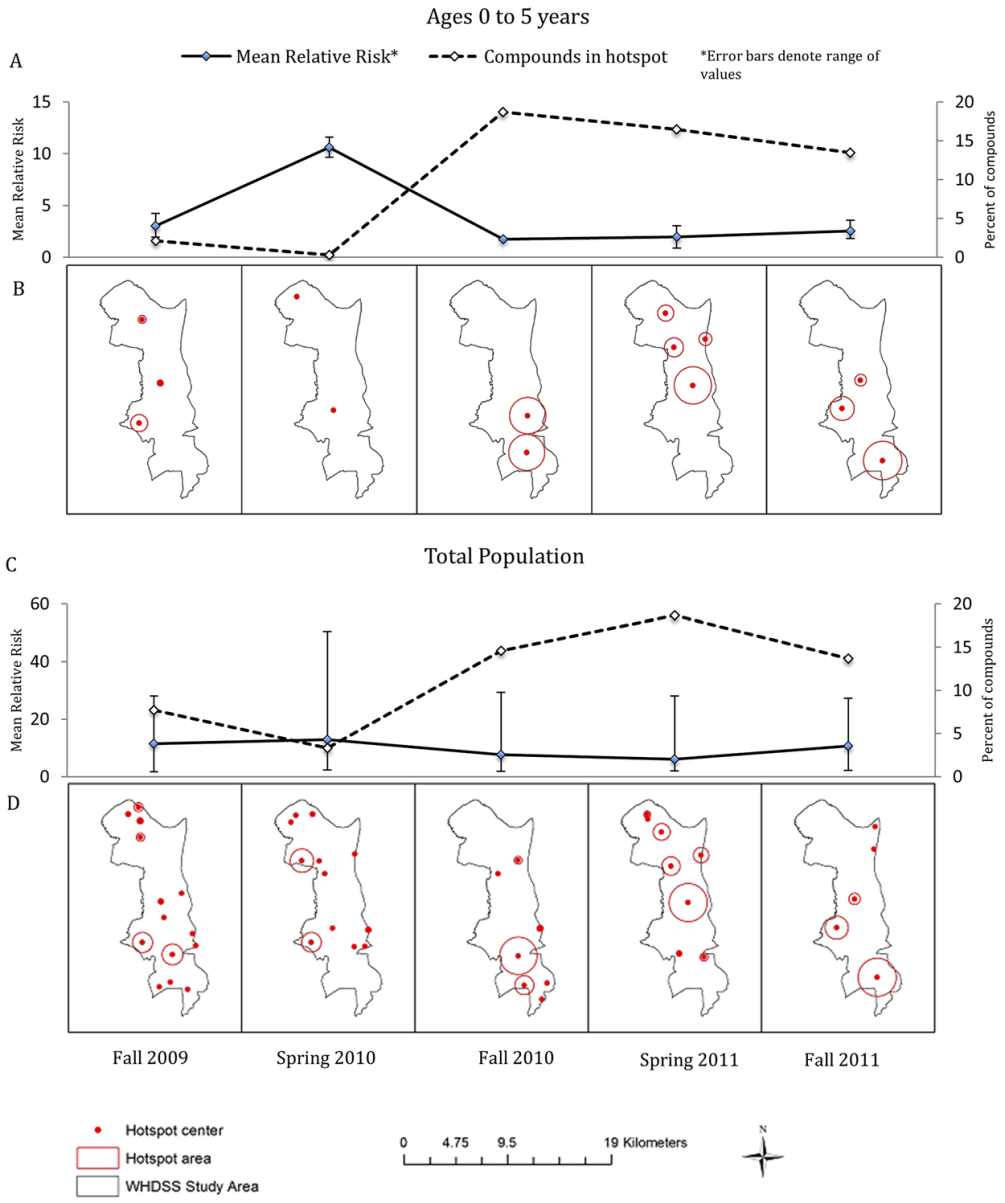
Hotspot location and size (**B**,**D**) and mean relative risk with percent of compounds in hotspots (**A**,**C**) by data collection period. Panels A and B are for compounds with children ages 0–5 years. Panel C and D show the total population. Spring refers to January 1 – June 30; Fall refers to July 1 – December 31. Maps created using ArcMap 10.2.2 (http://www.esri.com/arcgis/about-arcgis).

Between 2–4 hotspots and 5–14 hotspots per 6-month interval were identified in the 0 to 5 year olds and the total population, respectively (Fig. 1). In a given interval, the percentage of compounds in a hotspot was 10.2% (range: 0.3% − 18.6%) and 11.6% (range: 3.3% − 18.7%) on average for age 0 to 5 and total population samples, respectively. Relative risk within a hotspot ranged from 1.7 to 12.6 in the 0 to 5 year olds and 1.7 to 50.5 in the total population sample.

Hotspot number, location, and size varied from interval to interval. Overall, 34.7% of compounds were located in a hotspot in only a single interval while 47.9% were never in a hotspot. 15.9% of compounds were located in a hotspot in two intervals, but only 1.54% in three or more intervals. The smallest hotspot for the younger population was 120 meters but the smallest hotspot in the total population was just a single compound. In both the total population and the age 0 to 5 population, the maximum hotspot size was 2 km.

### Role of static variables in hotspot membership

Risk of membership in a hotspot at any point during the study was investigated as a function of temporally-constant socio-demographic and geographic factors (Table 3). Confounder variables, particularly TFA variables correlated with underlying risk of being in a hotspot (Figure S1). Not surprisingly given the dynamic nature of hotspots, other static predictors had relatively small effects on the risk of being in a hotspot. Distance to a health facility had the highest magnitude of effect on hotspot membership for the total population (associated risk increase of 12.0% per 1.2 km of distance) while distance to a river was most important for age 0 to 5 (associated risk increase of 15.0% per 1.45 km of distance).

**Table 3:** Modified Poisson Regressions of compound ever being located in a hotspot on selected static covariates.

VARIABLES	Age 0 to 5 Estimate Risk Ratio	Total population Estimate Risk Ratio
Compound population (maximum)^2^	0.97	**0**.**94**
	(0.95–1.00)	**(0**.**92–0**.**97)**
Household head or spouse in compound Farming	0.99	**1**.**08**
	(0.92–1.06)	**(1**.**02–1**.**14)**
Number of cattle owned	0.98	0.94
	(0.91–1.05)	(0.85–1.05)
Number of sheep/goats owned	**1**.**06**	**1**.**04**
	**(1**.**04–1**.**08)**	**(1**.**03–1**.**06)**
Number of poultry owned	0.94	1
	(0.79–1.11)	(0.87–1.15)
Acres of land owned by members of compound^2^	1	0.99
	(0.98–1.03)	(0.97–1.01)
Compound uses piped water	1.05	1
	(0.93–1.18)	(0.88–1.13)
Compound uses spring water	1.02	0.9
	(0.90–1.15)	(0.79–1.02)
Compound uses well water	0.95	0.91
	(0.84–1.07)	(0.80–1.03)
Compound uses other water source	1.03	1.02
	(0.91–1.17)	(0.89–1.16)
Distance to river^2^	**1**.**15**	**1**.**05**
	**(1**.**11–1**.**20)**	**(1**.**02–1**.**08)**
Distance to road^2^	**0**.**89**	0.97
	**(0**.**86–0**.**92)**	(0.94–1.00)
Distance to health facility^2^	**1**.**07**	**1**.**12**
	**(1**.**02–1**.**12)**	**(1**.**08–1**.**16)**
Constant	0.341	0.391
	(0.296–0.393)	(0.341–0.449)
Observations	6,160	7,682

^1^Regressions adjusted for number of rounds a compound was NOT in the sample and selected temporal Fourier analyzed (TFA) MODIS variable.

^2^Continuous value standardized for analysis.

**Figure 2:**
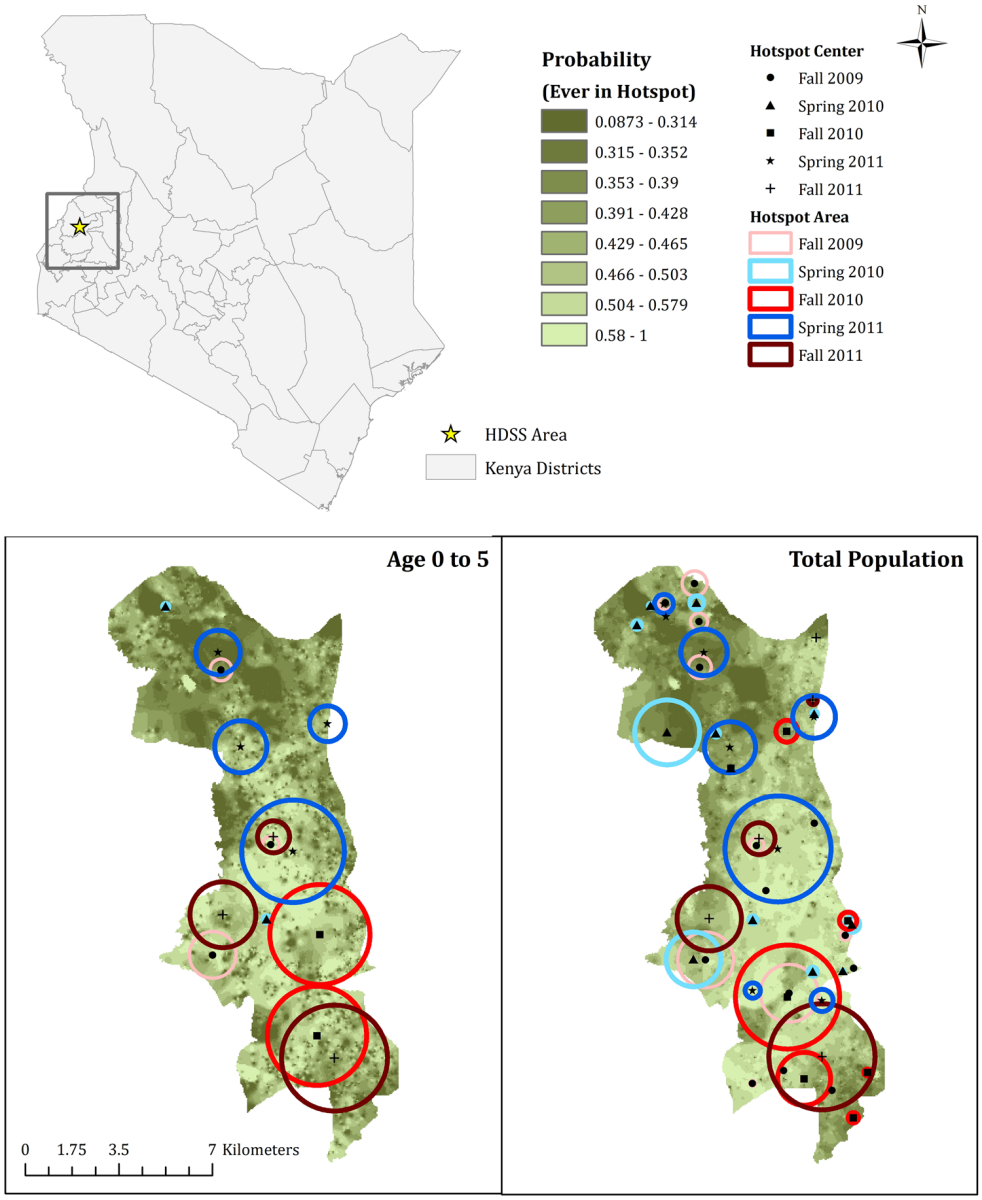
Study area location and modified Poisson model-fitted values for ever being located in a hotspot on static covariates and environmental confounders, by group. Maps were created using ArcMap 10.2.2 (http://www.esri.com/arcgis/about-arcgis).

In terms of non-spatially derived characteristics, farming had the highest magnitude effect on hotspot risk in the total population, while number of sheep/goats increased the risk of hotspot membership for children under five years. Using these static covariates, we describe a probability surface for hotspot membership for the study area (Fig. 2). Probability ranges from 0.1 to 1 and are generally higher in the southern region. There is good agreement between the probability maps for children and the total population. However, hotspots in areas of low predicted probability can clearly be seen indicating a set of covariates that cannot completely explain hotspot location.

In the total population sample, we found that there was dependence between the outcome and the probability of missing household characteristics, however, when we adjusted regressions using IPW, we found no meaningful change in parameter estimates in the magnitude or direction of effect.

### Temporal variability of hotspots

**Figure 3:**
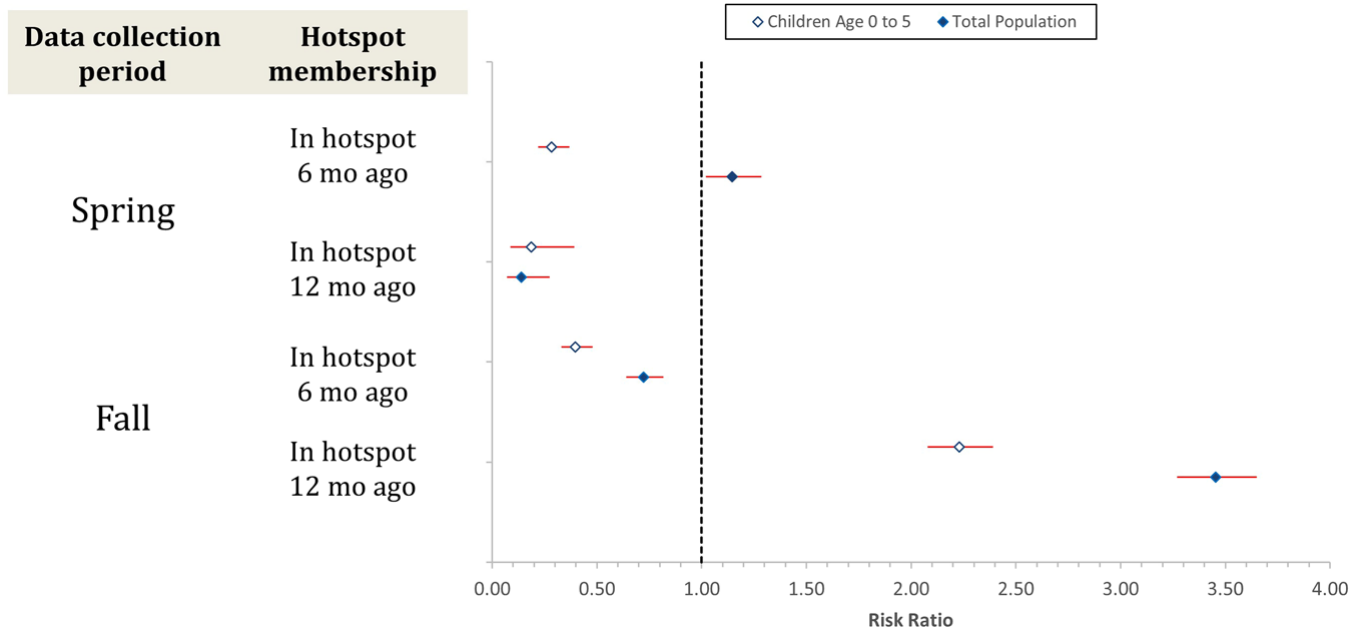
Risk ratio with 95% CI for hotspot membership by sample and season.

To better understand stability of hotspot location over time, we analyzed hotspot membership in a 1 and 2 interval lagged outcome model, including an interaction effect to account for differences in lag effects by spring vs. fall interval (Fig. 3, Supplemental Table 1). We found significant lagged effects of hotspot membership which were indeed modified by timing of the current interval. In the population of children age 0 to 5, we found that, regardless of timing, a compound was not likely to be in a hotspot in contiguous intervals with the reduction in risk larger in the spring (Spring RR = 0.28, 95% CI: 0.22–0.37; Fall RR: 0.7740, 95% CI: 0.363–0.9348). In the total population, the risk of being in a hotspot in two contiguous intervals depended on season with an increased risk of being in a hotspot in the spring if a compound was in a hotspot in the previous 6-month interval (RR = 1.15, 95% CI: 1.02–1.29) and a decreased risk of being in a hotspot in the fall if a compound was in a hotspot in the previous 6-month interval (RR = 0.81, 95% CI: 0.70–0.94).

In both populations, the effect of having been in a hotspot one year prior depended strongly on whether the timing of the interval within the year. Households were more likely to be in a hotspot in the fall interval if they had been in a hotspot two intervals prior by a factor of 1.68 (95% CI: 1.54–1.84) in children age 0 to 5 and 1.77 (95% CI: 1.66–1.89) in the total population, while the opposite was true for annual cycles starting in the spring.

Taken together, these results suggest that hotspots move in space and that they may be qualitatively different in early and late seasons. Over the course of our study period, fall hotspots were more stable and more often recurred in the same locations in subsequent late intervals. Spring hotspots were less likely to occur in the same location as the previous interval, or the previous year.

### Population dynamics and hotspot location

Human migration and births had a significant effect on hotspot location and these effects were similar for children age 0 to 5 and the total population (Table 4). In-migrations from other WHDSS households that had not been located in a hotspot in the previous interval and migration from urban and rural areas outside of the WHDSS area were associated with increased risk of hotspot membership. In-migration from a WHDSS household that had been in a hotspot in the previous interval was associated with a 55% reduced risk of current location in a hotspot for the total population, though the wide confidence interval suggests this estimated effect was quite imprecise (95% CI: 8% − 78%). Compound population itself was not associated with a change in risk of location in a hotspot in either group. However, in-migration always increased the risk of being in a hotspot except in-migration from other WHDSS hotspots. Migrants from within the WHDSS had an overall larger effect on hotspot risk than migrants from outside the WHDSS.

**Table 4:** Modified Poisson Regressions of compound in a hotspot on dynamic covariates^1^.

**Variable**	**Age 0 to 5 Estimate Risk Ratio**	**Total Population Estimate Risk Ratio**
Number of newborns	0.98	0.99
	(0.90–1.06)	(0.91–1.08)
Number of in-migrations from hotspot	**0**.**33**	**0**.**45**
	**(0**.**12–0**.**89)**	**(0**.**22–0**.**92)**
Number of in-migrations from non-hotspot	**1**.**16**	1.13
	**(1**.**03–1**.**32)**	(0.99–1.30)
Number of in-migrations from urban area	**1**.**17**	**1**.**19**
	**(1**.**07–1**.**28)**	**(1**.**09–1**.**30)**
Number of in-migrations from rural area	**1**.**11**	**1**.**11**
	**(1**.**04–1**.**20)**	**(1**.**03–1**.**19)**
Compound population^2^	0.99	**0**.**95**
	(0.96–1.02)	**(0**.**92–0**.**97)**
Constant^3^	0.09	0.12
	(0.08–0.09)	(0.11–0.12)
Observations	22,608	31,805
Number of groups	6,382	8,218

^1^Regressions adjusted for Fall vs. Spring. ^2^Continuous value standardized for analysis. ^3^Interpretation of this parameter should be as a risk rather than a risk ratio.

## Discussion

In this study, we examine the temporal stability of malaria hotspots in a large, population-based open cohort in western Kenya. In a population of 64,000 people, we find that hotspots move in space across six-month intervals and rarely recur in the same position. Only a small proportion of households were located in a hotspot more than once in the three-year study period. Falling within a hotspot in one six-month interval substantially reduced the risk of belonging to a hotspot in the following interval. The same is true one year later, but only if the household was in a hotspot in a spring interval (corresponding to the long rains). However, those who were in a fall hotspot (during the short rains) had a nearly 2-fold higher risk of being in a hotspot one year later during the following fall. The difference in the six-monthly versus annual patterns is modified by season and suggests a qualitative difference between spring and fall hotspots.

Human movement, particularly migration into a household, played a significant role in risk of hotspot membership. Human movement from areas with differing levels of exposure and importation of varying parasite genotypes have been identified as risk factors in malaria transmission in Africa^8,32,33^. However, this is the first time that human population dynamics have been implicated in local foci of elevated transmission. The magnitude and direction of the effect was specific to the origin of the migration event and was independent of total household size. Local migration within the study area was most strongly predictive of hotspot membership. Individuals migrating from nearby hotspots *reduced* the risk of hotspot membership in the next six-month interval, but migration from non-hotspots had the opposite effect. Based on this information, it seems less likely that migration is responsible for parasite importation and more likely that a migration event from a non-hotspot brings a susceptible individual into an area of elevated exposure, thus increasing the risk of a clinical episode. Conversely, a migrant from a recent hotspot is more likely to have some elevated immunity thus decreasing the risk of a new clinical episode. This is supported by the observation that urban migrants increase risk of hotspot membership. It is interesting that rural migrants also increase the risk of hotspot membership. Although a rural area may not be endemic (for example, coming from the highlands), rural areas are overall likely to have higher malaria transmission than urban areas. We cannot rule out the possibility of a migrant from outside the study area, whether rural or urban, importing an antigenically distinct parasite for which local immunity is lower thus increasing the risk of a local hotspot.

Although specific static covariates were correlated with elevated risk of hotspot membership, the magnitude of the association was modest. Amongst the static variables, geographic location with respect to the road, the river, and a health facility were most strongly correlated with risk of being in a hotspot. Consistent with other studies, we also find that hotspots are associated with a mix of other static risk factors such as environmental conditions^9,34,35^, altitude^4,35^, and participation in farming^8,36^.

The dynamic nature of hotspots in our cohort has major implications for hotspot targeting. At least one other study has identified spatially dynamic hotspots^15^ and one documented hotspots emerging as transmission declined^14^. Bejon *et al.*^9^ found that hotspots of symptomatic, febrile malaria were more mobile in space than foci of asymptomatic infection. This is probably because the foci for asymptomatic carriage of malaria parasites arise from malaria endemic areas with perennial transmission, and as a result have malaria reservoir among infected individuals in excess of 1000-parasite/μℓ blood required to trigger gametogony^37^. However, symptomatic and febrile malaria hotspots are located in foci where malaria infection are transient and occur mainly as a result of mosquito succession following the onset and progression of seasonal rains^38^. These rains are usually non-uniform over a geographical area and malaria vector breeding will be spatially and temporally variable as the rains progress. It remains to be determined whether hotspots defined on larger geographic scales are as dynamic.

Our study has several limitations which must be weighed when interpreting the results. First, our outcome of symptomatic malaria is defined by self-report. Although it is distinguished from unknown febrile illness and other common morbidities in the dataset, it is likely that malaria is over-reported and therefore our definition has high sensitivity but low specificity. There is also some concern that those experiencing fever may assume malaria is the cause or may refer to any fever with the same terminology as they do malaria^39^. However, morbidity reports from the WHDSS include other causes of fever (e.g. otitis media, typhoid fever, other infections) as well as non-specific fever (ICD 10CM R50.9), reported both by individuals who seek treatment in health facilities and those who self-medicate, indicating that both facilities and individuals are distinguishing between malaria and other causes of fever (37% of non-specific fever is reported by individuals who sought treatment as health facilities). Furthermore, self-reported malaria cases show the seasonality expected of malaria (see Supplemental Fig. 2). Other common causes of pediatric fever, such as influenza, have shown different seasonality^40^. Even in the presence of significant over-reporting of malaria, our identification of hotspots will not be biased unless the over-reporting varies spatially.

Recall bias may also have affected the accuracy of the data^41–44^, although it is unlikely to be more problematic within hotspots than outside and therefore unlikely to have influenced the detection of hotspots. Second, the period of data collection, 2.5 years, while longer than many previous studies, is a relatively short analytical time frame and limits our ability to describe the long-term mobility or annual patterns of hotspot location. Finally, there are undoubtedly many relevant static and dynamic factors that we were not able to incorporate into our model, such as bednet use and proximity to mosquito habitats.

This is the first study of which we are aware that uses a large, longitudinal census population to explore hotspot location at the level of residential location of households. Our study population is more than ten-times larger than previous longitudinal hotspot analyses. This allowed us to look at malaria incidence for all ages in a contiguous geographic area rather than a sub-sample of geographic locations or a specific age group^1,9,14^. Furthermore, morbidity was recorded from all residents rather than from a sample reporting to a health facility^1^. The census structure also allows individuals to enter or leave the sample over time, incorporating true population dynamics into the malaria hotspot risk model. The longitudinal structure of the data allowed us to not only to track individuals and households over time and space, but also allowed us to compare hotspot location between seasons and years. Our findings are highly relevant to studies exploring hotspot interventions and highlight the challenge of predicting hotspot locations. More research is needed to understand the factors that determine hotspot location, with special emphasis on those that can be measured dynamically on appropriate spatial and temporal scales.

### Data Availability

Original datasets contain identifiable information on subjects and must be requested separately from the WHDSS through Moi University. De-identified analysis datasets and analysis programs are available through the corresponding author.

## Electronic supplementary material

Supplementary Materials
