# Supplementary material for: Dynamic malaria hotspots in an open cohort in western Kenya
Source: Sci Rep. 2018 Jan 12;8:647. doi: 10.1038/s41598-017-13801-6 (PMC5766583; doi:10.1038/s41598-017-13801-6)
Supplement: Supplementary file 1 — Supplementary Materials [file 41598_2017_13801_MOESM1_ESM.pdf]

Dynamic malaria hotspots in an open cohort in western Kenya

Alyssa Platt[1,2]*, Andrew A. Obala[3], Charlie MacIntyre[1,4], Barasa Otsyula[3], Wendy Prudhomme O'Meara[1,2,5]

[1]Duke Global Health Institute, Durham, North Carolina, United States of America, [2]Department of Biostatistics and Bioinformatics, Duke University, North Carolina, United States of America,[3]College of Health Sciences, Moi University, Eldoret, Kenya, [4]Campbell University School of Osteopathic Medicine, [5]Department of Medicine, Duke University, Durham, North Carolina, United States of America

*Correspondence to alyssa.platt@duke.edu

Supplemental File 1: Variable creation and model selection for environmental confounder variables, detailed methods

Topographic Wetness Index

NASA Advance Spaceborne Thermal Emission and Reflection Radiometer (ASTER) Global Digital Elevation Model (GDEM) at 30m resolution was extracted for the HDSS area. A topographical wetness index was created by combining calculations of flow direction, flow accumulation and slope parameters in ArcGIS 10.2[1]

TFA Analyzed MODIS Data

MODIS data are available at a temporal resolution of 8 or 16 days and spatial resolutions of 250, 500, and 1000m. Data for daytime and nighttime Land Surface Temperature (LST) (MODIS File: MOD11A2) were extracted at 8-day, 1 km resolution. Enhanced Vegetation Index (EVI) and Middle Infrared reflectance (MIR) were extracted at the 16 day, 250m resolution (MODIS File: MOD13Q1) for each compound in the study area using linear interpolation performed in ArcGIS 10.2. Linear interpolation was performed to replace missing values or values out of the plausible range within each time series for each compound. Spline interpolation was then used to convert the 8 and 16 day resolution to 2.5 day resolution. Each resulting dataset was then processed using temporal Fourier analysis (TFA) using the HarmonicRegression package in R software (version 3.1.2) [2] to obtain parameters describing the seasonal variation in each variable. Mean, minimum, maximum, amplitude, phase, and sum of squared residuals were extracted for annul, biannual, and triannual cycles [3].

Variable selection

We employed a process of variable selection in order to reduce the dimensionality of and eliminate highly correlated environmental variables. In order to include only the most relevant, we first used least absolute shrinkage and selection operator (LASSO) regressions for preliminary reduction of variables[4] using an outcome of if a compound was ever in a hotspot. The remaining variables were entered into modified Poisson regressions[5] and variables that were not statistically significant were eliminated from the regression. Akaike and Bayesian Information Criterion (AIC/BIC) were used to confirm that model fit improved upon elimination of non-significant parameter estimates [6]. The remaining variables were entered into a correlation matrix. All variables with correlations greater in magnitude than 0.70 [7] were compared via AIC. The variable in the correlated pair that was most related to the outcome of hotspot location was kept and the other discarded. A second modified Poisson regression was run using the remaining variables, followed by elimination of non-significant parameter estimates, with improved fit confirmed via AIC (as above). Finally, relative risks with the remaining variable regressions were ordered by magnitude and variables with the smallest magnitude of RR were eliminated one by one until their elimination failed to improve model fit (again, evaluated via AIC). This resulted in 21 selected variables (Supplemental Figure 1).

References

1. Sørensen R, Zinko U, Seibert J. On the calculation of the topographic wetness index: evaluation of different methods based on field observations. *Hydrology and Earth System Sciences Discussions* 2005; **2**: 1807-34.
2. Westermark PO. Harmonic Regression to One or more Time Series. 1.0 ed: CRAN; 2015.
3. Scharlemann JP, Benz D, Hay SI, et al. Global data for ecology and epidemiology: a novel algorithm for temporal Fourier processing MODIS data. *PLoS ONE* 2008; **3**: e1408.
4. Tibshirani R. Regression shrinkage and selection via the lasso. *Journal of the Royal Statistical Society Series B (Methodological)* 1996: 267-88.
5. Zou G. A Modified Poisson Regression Approach to Prospective Studies with Binary Data. *American Journal of Epidemiology* 2004; **159**: 702-6.
6. Akaike H. A new look at the statistical model identification. *Automatic Control, IEEE Transactions on* 1974; **19**: 716-23.
7. Chiulli RM. *Quantitative analysis: An introduction*: CRC Press; 1999.

Supplementary Table 1. Modified Poisson regression models[1] of the relationship between current hotspot location and lagged location, by season

Variables	Age 0 to 5		Total Population	
	Last Season	Last Year	Last Season	Last Year
	Risk Ratio	Risk Ratio	Risk Ratio	Risk Ratio
Fall[2]	**1.95**	**0.82**	**1.37**	**0.60**
	**(1.81 - 2.11)**	**(0.76 - 0.89)**	**(1.29 - 1.46)**	**(0.56 - 0.64)**
In a hotspot (lagged)[3]	**0.28**	**0.19**	**1.15**	**0.14**
	**(0.22 - 0.37)**	**(0.09 - 0.39)**	**(1.02 - 1.29)**	**(0.07 - 0.27)**
Fall x lagged hotspot[3,4]	**1.40**	**12.01**	**0.63**	**24.75**
	**(1.00 - 1.95)**	**(5.68 - 25.42)**	**(0.53 - 0.75)**	**(12.64 - 48.45)**
Constant[4]	0.10	0.19	0.12	0.21
	(0.10 - 0.11)	(0.18 - 0.20)	(0.11 - 0.12)	(0.20 - 0.22)
**Contrasts**				
Lagged effect (when Spring)[2,3]	**0.284**	**0.21**	0.98	**0.15**
	**(0.22 - 0.37)**	**(0.10 - 0.41)**	(0.86 - 1.12)	**(0.08 - 0.28)**
Lagged effect (when Fall)[3]	**0.77**	**1.68**	**0.81**	**1.77**
	**(0.63 - 0.93)**	**(1.54 - 1.84)**	**(0.70 - 0.94)**	**(1.66 - 1.89)**
Lagged effect (Fall – Spring)[3]	**2.73**	**8.2**	0.83	**12.19**
	**(1.98 - 3.79)**	**(4.15 - 16.20)**	(0.68 - 1.00)	**(6.41 - 23.20)**
Observations	22,155	16,554	31,120	23,433
Number of groups	6,366	6,099	8,201	8,101

[1]Correlation over time between repeated measurements made on the same household are accounted for using generalized estimating equations (GEE) with exchangeable working correlation and robust standard errors

[2] Spring refers to January 1 through June 30; Fall refers to July1 through December 31 of any year

[3]For children age 0 to 5, lagged outcome could include age 0 to 5 hotspots as well as total population hotspots.

[4]Interpretation of this parameter should be as a ratio of risk ratios rather than simply a risk ratio

[5]Interpretation of this parameter should be as a risk rather than as a risk ratio

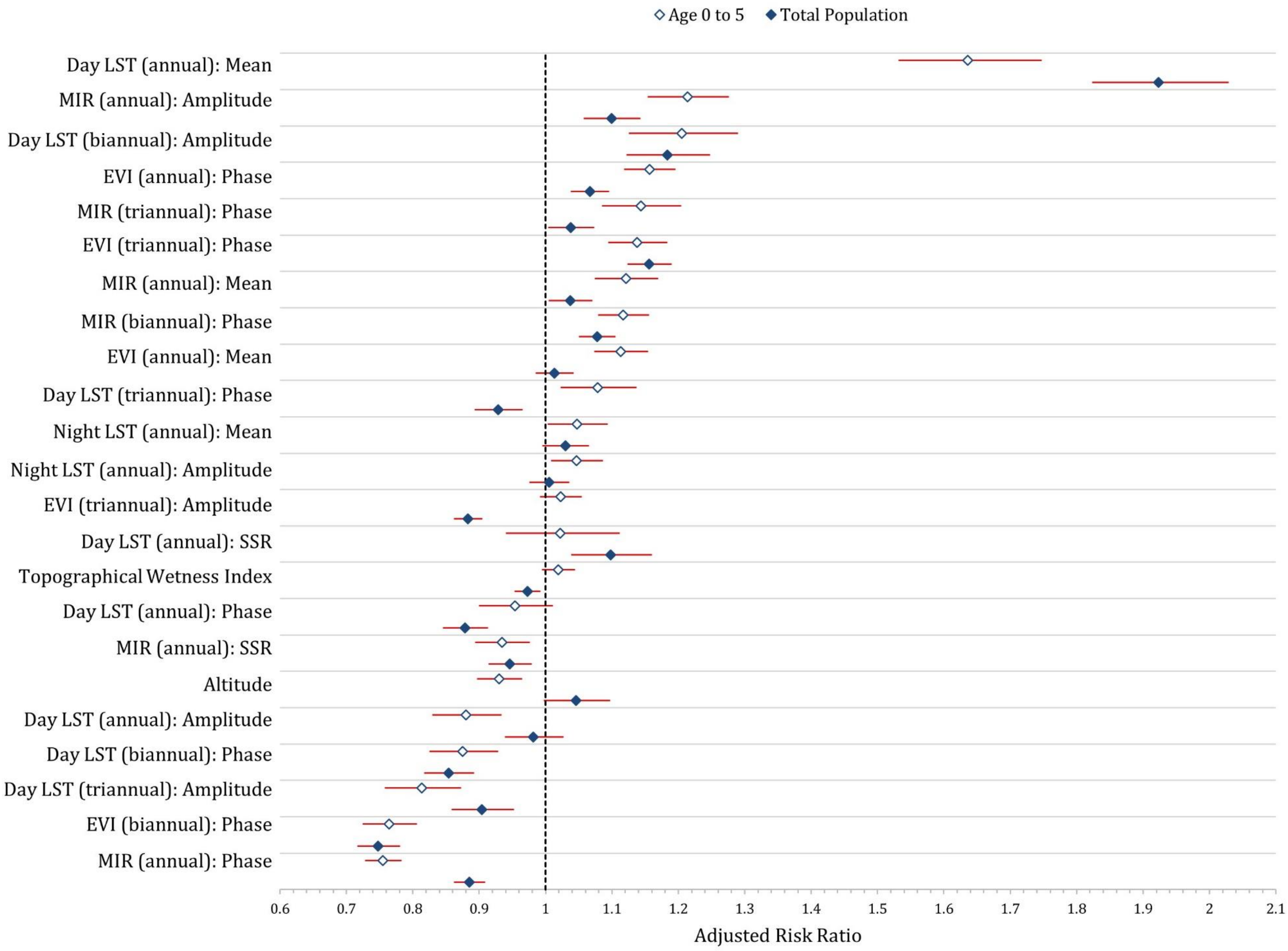
Age 0 to 5
Total Population
Day LST (annual): Mean
MIR (annual): Amplitude
Day LST (biannual): Amplitude
EVI (annual): Phase
MIR (triannual): Phase
EVI (triannual): Phase
MIR (annual): Mean
MIR (biannual): Phase
EVI (annual): Mean
Day LST (triannual): Phase
Night LST (annual): Mean
Night LST (annual): Amplitude
EVI (triannual): Amplitude
Day LST (annual): SSR
Topographical Wetness Index
Day LST (annual): Phase
MIR (annual): SSR
Altitude
Day LST (annual): Amplitude
Day LST (biannual): Phase
Day LST (triannual): Amplitude
EVI (biannual): Phase
MIR (annual): Phase
0.6
0.7
0.8
0.9
1
1.1
1.2
1.3
1.4
1.5
1.6
1.7
1.8
1.9
2
2.1
Adjusted Risk Ratio

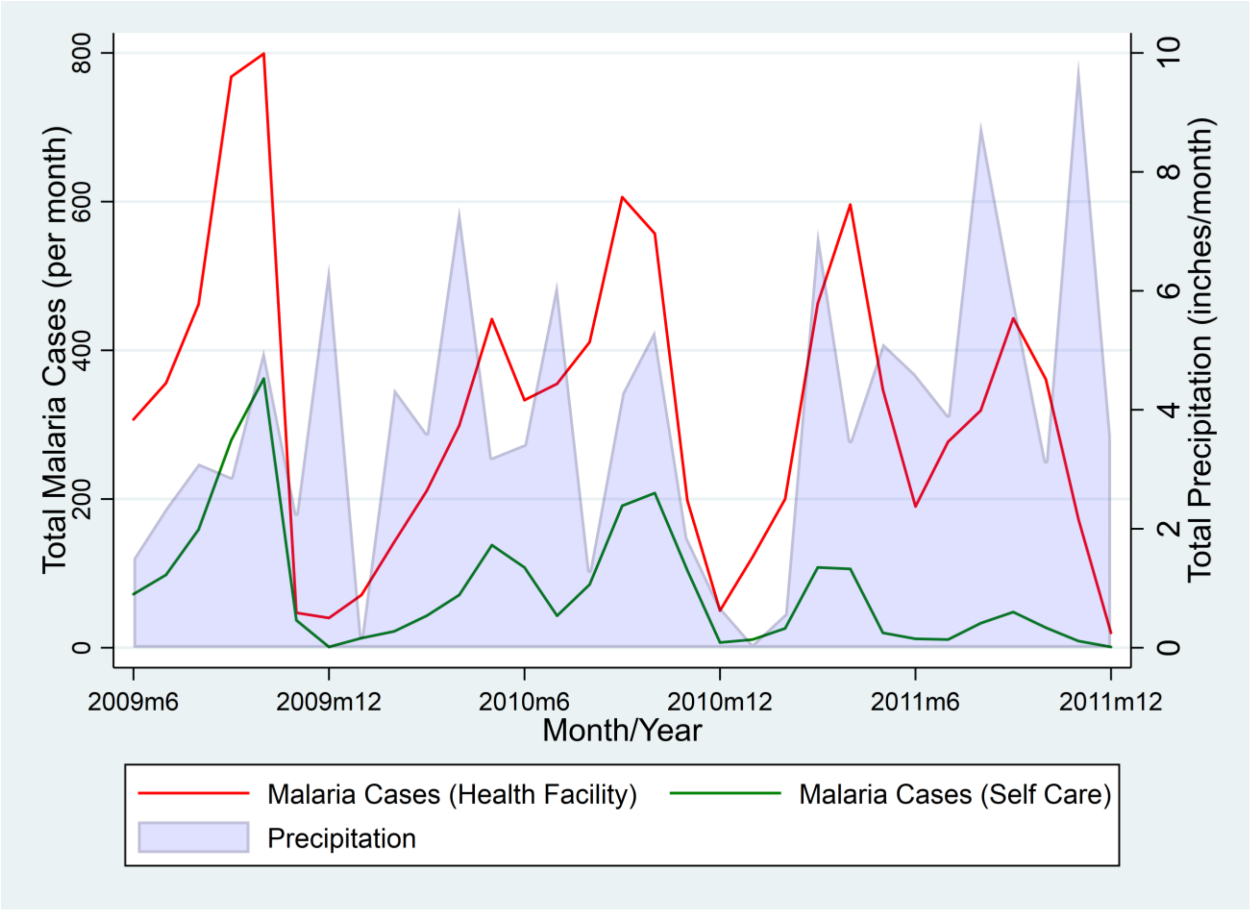

Total Malaria Cases (per month)
800
600
400
200
0
Total Precipitation (inches/month)
10
8
6
4
2
0
2009m6
2009m12
2010m6
2010m12
2011m6
2011m12
Month/Year
Malaria Cases (Health Facility)
Malaria Cases (Self Care)
Precipitation

Figure Legends

Supplemental Figure 1. Adjusted risk ratios with 95% CI for environmental confounders from modified Poisson regressions of ever being in a hotspot on static household covariates and environmental confounders, by group. LST = Land surface temperature; MIR = Middle Infrared Reflectance; EVI = Enhanced Vegetation index.

Supplemental Figure 2. Total monthly malaria cases treated at health facilities vs. self-care over WHDSS study period; with total monthly precipitation.
